# Exercise Improves Endothelial Function Associated with Alleviated Inflammation and Oxidative Stress of Perivascular Adipose Tissue in Type 2 Diabetic Mice

**DOI:** 10.1155/2020/8830537

**Published:** 2020-12-26

**Authors:** Jinju Wang, Venkata Polaki, Shuzhen Chen, Ji C. Bihl

**Affiliations:** ^1^Department of Biomedical Sciences, Joan C Edwards School of Medicine, Marshall University, Huntington, WV 25755, USA; ^2^Department of Pharmacology and Toxicology, Boonshoft School of Medicine, Wright State University, Dayton, OH 45435, USA

## Abstract

Perivascular adipose tissue (PVAT), a type of adipose tissue that surrounds the blood vessels, has been considered an active component of the blood vessel walls and involved in vascular homeostasis. Recent evidence shows that increased inflammation and oxidative stress in PVAT contribute to endothelial dysfunction in type 2 diabetes (T2D). Exercise is an important nonpharmacological approach for vascular diseases. However, there is limited information regarding whether the beneficial effects of exercise on vascular function is related to the PVAT status. In this study, we investigated whether exercise can decrease oxidative stress and inflammation of PVAT and promote the improvement of endothelial function in a T2D mouse model. Diabetic db/db (5-week old) mice performed treadmill exercise (10 m/min) or keep sedentary for 8 weeks. Body weight, fasting blood glucose levels, glucose, and insulin tolerance were determined. The cytokines (IL-6, IL-10, IFN-*γ*, and TNF-a) and adiponectin levels, macrophage polarization and adipocyte type in PVAT, oxidative stress, and nitric oxide (NO) expression in the vascular wall were evaluated. The adhesion ability of primary aorta endothelial cells was analyzed. Our data showed that (1) diabetic db/db mice had increased body weight and fasting blood glucose level, compromised glucose tolerance, and insulin sensitivity, which were decreased/improved by exercise intervention. (2) Exercise intervention increased the percentage of multilocular brown adipocytes, promoted M1 to M2 macrophage polarization, associating with an increase of adiponectin and IL-10 levels and decrease of IFN-*γ*, IL-6, and TNF-a levels in PVAT. (3) Exercise decreased superoxide production in PVAT and the vascular wall of diabetic mice, accompanied with increased NO level. (4) The adhesion ability of aorta endothelial cells to leukocytes was decreased in exercised db/db mice, accompanied by decreased intercellular adhesion molecule 1 (ICAM-1) and vascular cell adhesion molecule 1 (VCAM-1) expressions. Of interesting, coculture with PVAT-culture medium from exercised db/db mice could also reduce ICAM-1 and VCAM-1 expressions in primary endothelial cells. In conclusion, our data suggest that exercise improved endothelial function by attenuating the inflammation and oxidative stress in PVAT.

## 1. Introduction

Diabetes is a group of chronic diseases characterized by hyperglycemia. Over the last several decades, the global incidence and prevalence of diabetes mellitus have increased significantly. In the United States alone, the incidence of type 2 diabetes (T2D) has increased by 40% during the past decades. Diabetes mellitus is not merely a disorder of carbohydrate metabolism, but a cause of vascular disease affecting nearly all blood vessels. Indeed, vascular complications are the most serious manifestations responsible for most of the morbidity, hospitalizations, and death that occur in patients with diabetes mellitus, despite several clinical advances that have been made. Therefore, novel strategies capable of protecting diabetic vascular function are urgently needed.

In addition to the widespread consumption of a high fat/sucrose diet, lacking regular physical activity contributes to the progression of T2D and associated vascular diseases. Exercise has beneficial effects on several cardiovascular risk factors such as diabetes and hypertension [1]. The Diabetes Prevention Program showed that regular physical activity intervention could significantly reduce the risk of developing diabetes in high-risk individuals [2]. Our group has demonstrated that exercise has beneficial effects on ischemic stroke [3]. Exercise training has also been shown to improve endothelial function in subjects with prehypertension or hypertension [4]. However, the underlying mechanism of how exercise-induced vascular benefits have not been fully answered.

Adipose tissue is a complex set of cells and could be classified into white and brown adipose tissue. Perivascular adipose tissue (PVAT) is a type of adipose tissue that surrounds vessels to provide mechanical protection of vascular structures. It represents around 3% of the total body adipose tissue mass [5]. Previous studies have revealed that PVAT has different characteristics in different anatomic locations. For instance, PVAT surrounding the abdominal aorta displays characteristics of white adipose tissue, whereas thoracic aorta PVAT contains multilocular lipid droplets which are typical characteristics of brown adipose tissue [6]. In addition to the structural role, it is increasingly being appreciated that PVAT plays other roles in vascular function including the secretion of metabolically active adipokines, adiponectin, and adipocyte-derived relaxing factors to regulate vascular tone under physiological condition [7]. The latest study reveals that PVAT could assist arterial stress relaxation [8], indicating PVAT is an essential player in maintaining vascular health.

In obesity and diabetes, the properties of PVAT appear to be altered and PVAT becomes dysfunctional [9]. It expands and becomes a mixture of white and brown adipose tissue [10]. Meanwhile, the protective factors secretion is reduced and the PVAT becomes inflamed with the accumulation of inflammatory cells and alteration in secreting inflammatory cytokines [11]. These studies suggest that PVAT might serve as a mechanistic link between metabolic syndrome and vascular diseases such as atherosclerosis [12]. Indeed, there is increasing interest in investigating the potential roles of PVAT in vascular dysfunction in metabolic diseases such as obesity and T2D. Animal studies have revealed that overproduction of oxidative reactive species (ROS) and increased inflammation in PVAT might be linked to vascular dysfunction in obesity [13, 14]. In the patient with T2D, the adiponectin level produced by PVAT of internal mammary arteries is correlated with the increment in nicotinamide adenine dinucleotide phosphate oxidase activity in these arteries [15]. Taken together, all of these findings suggest the involvement of PVAT in modulating vascular function.

Exercise has been shown to alleviate inflammation in adipose tissue in high-fat diet-fed mice through inducing a macrophage phenotype switch from M1- to M2-macrophages and preventing macrophage infiltration in the gonadal adipose tissue [16, 17]. To date, there is limited information regarding whether exercise can modulate the properties of PVAT and whether this contributes to exercise-induced benefits on endothelial function in T2D. In this study, we aimed to investigate whether treadmill exercise intervention can alleviate inflammation and oxidative stress of PVAT and thereby contribute to improving the vascular function in T2D mice.

## 2. Methods and Materials

### 2.1. Animals

Homozygous type 2 diabetic mice (db/db; 5-6 weeks old; Leprdb; background strain: C57BLKS/J) and heterozygous control mice (db/c, 5-6 weeks old; Leprdb; background strain: C57BLKS/J) were purchased from Jackson Laboratory. All animals were housed in an animal facility conditioned with 12-h : 12-h light/dark cycles and allowed free access to normal chow and water. A total of 96 mice were used in this study. Db/db mice conducted treadmill pre-training for 1 week and then were randomly divided into exercise (*n* = 32) and nonexercise (*n* = 32) groups. Age-matched db/c mice (*n* = 32) were used as controls for the study. All animals were euthanized at the age of 14 to 15 weeks old. Body weights were recorded weekly. All experimental procedures were approved by the Wright State University Laboratory Animal Care and Use Committee and were in accordance with the Guide for the Care and Use of Laboratory Animals issued by the National Institutes of Health.

### 2.2. Treadmill Exercise Intervention

Treadmill exercise intensity was based on previous studies showing exercise has beneficial effects on cardiovascular diseases with slight modification [3]. Briefly, all mice were acclimatized to run on a motorized rodent treadmill with an electric grid at the rear of the treadmill (Columbus Instruments, Columbus, OH) for 6 days before conducting the 8-week exercise intervention. During acclimation, treadmill duration was 5 m/min for 30 mins on day 1. Speed was then increased by 1 m/min, and time was increased by 10 mins on a daily basis to the target speed of 10 m/min and time for 60 mins. Then, the treadmill speed was set at 10 m/min for exercise intervention. A daily 60-min exercise was repeated for 8 weeks, 5 days per week.

### 2.3. Fasting Blood Glucose Test

For all mice, fasting blood glucose was monitored on the sixth day of every exercise week. In brief, on the fifth day of every exercise week, db/db exercised and nonexercised, as well as db/c mice, were fasted overnight (for 14-18 hrs) with water supply. On the next day, blood glucose was measured by commercial One Touch UltraSmart glucometer (Lifescan, Milpitas, CA) [18].

### 2.4. Glucose Tolerance Test (GTT)

For all mice, the GTT was tested on the next day after the last bout of exercising [19]. In general, after the last bout of exercise on the eighth week, mice fasted overnight with water supply for 14-18 hrs. On the next day, the glucose solution was injected intraperitoneally with a 27-gauge sterile needle. The concentration of glucose was 1 gram/kg of 20% pharmaceutical grade glucose [19]. After the injection, the mouse was returned to the home cage, and blood glucose levels at 30, 60, 90, and 120 min were measured by commercial One Touch UltraSmart glucometer (Lifescan, Milpitas, CA).

### 2.5. Insulin Tolerance Test (ITT)

ITTs were carried out on the next day after the last bout of exercising on the eighth week. All mice fasted with water supply for 5-6 hr on the day of the experiment [19]. After baseline blood glucose level was determined, each mouse received a 0.75 unit/kg i.p. injection of pharmaceutical-grade insulin. Blood glucose levels were then be measured at 30, 60, 90, and 120 min postinjection.

For repeated blood collection (GTT and ITT), the scab was removed from the tail nick so that additional cuts were not required. After testing, mice were provided ad libitum food.

### 2.6. Hematoxylin and Eosin (H&E) Staining

After an 8-week exercise, the thoracic aorta PVAT samples were collected from db/c mice, nonexercised, and exercised db/db mice. Tissue was fixed in 4% formalin and paraffin-embedded. Then, the tissue samples were sequentially cut into 5 *μ*m thickness sections and used for H&E staining according to the protocol we published [20]. The single droplet and multilocular adipocytes were counted under 5 different microscopic areas. Data from ten individual sections represented the data from one mouse.

### 2.7. Immunofluorescence Staining of PVAT

To determine whether exercise intervention could affect macrophage polarization in PVAT, the paraffin-embedded PVAT sections (10 *μ*m thickness) were deparaffinized and rehydrated prior to antigen unmasking by boiling in 10 mM sodium citrate (pH 6.0) for 10 mins. Sections were cool down for 30 mins, then washed with distilled water for twice, 5 mins of each. After permeabilization with permeabilization solution (3% BSA with 0.1% triton-100 in PBS) for 1 hr, sections were incubated with primary antibodies rat anti-F4/80 (Abcam, 8 *μ*g/ml), mouse anti-CD86 (Abcam, 4 *μ*g/ml), or rabbit anti-CD206 (Abcam, 4 *μ*g/ml) for overnight at 4°C. Followed by three times wash with PBS, sections were probed with FITC- or Cy3-conjugated secondary antibody for 2 hrs at RT. Cell nucleus was count stained with DAPI for 5 mins at room temperature. Images were taken under a fluorescence microscope (Nikon, Eclipse E600). F4/80+CD86+ cells were considered as M1 macrophages, and F4/80+CD206+ cells were considered as M2 macrophages. Cell numbers were averaged from 5 different fields. Data from ten individual sections represented the data from one mouse.

### 2.8. Flow Cytometry Analysis of the Macrophage Phenotypes in PVAT

On the next day of the last bout of exercise, PVAT from the three groups were collected and digested by collagenase for flow cytometry analysis as described with slight modification [21]. In brief, the collected PVAT was cut into small pieces and rinsed with PBS before digestion with 2 mg/ml collagenase I (Sigma) for 90 mins. Floating adipocytes were separated by centrifugation at 600 g for 10 mins. The pellets were resuspended with 100 *μ*l FACS buffer (PBS with 1 mM EDTA, 25 mM HEPES, and 1% heat-inactivated fetal bovine serum (FBS) and incubated with primary antibodies: FITC-conjugated anti-F4/80, PE-conjugated anti-CD206, and APC-conjugated anti-CD86. The relative antibody isotypes were used. All antibodies were purchased from eBioscience. Samples were analyzed by flow cytometry (Accuri C6 flow cytometer). Cells were first gated on F4/80+ cells. Then, the double-positive cells within the gate were analyzed.

### 2.9. Reactive Oxidative Stress Measurement on PVAT and Aorta

After the last bout of exercise in the eighth week, aortic segments with PVAT were collected, embedded in freezing medium (Tissue-Tek), and sectioned by using a cryostat. Frozen sections (20 *μ*m thickness) were incubated with 50 *μ*M dihydroethidium (DHE, Thermo Fisher) at 37°C for 30 mins in the dark. Images were obtained with a fluorescence microscope (Nikon, Eclipse E600). Quantitation of ROS was determined by comparing the mean intensities of 5 areas in each section of the red fluorescence using the ImageJ software (NIH).

### 2.10. Vascular Nitric Oxide (NO) Detection

NO production in the thoracic aorta was measured by using the NO-sensitive fluorescent dye 4,5-diaminofluorescein diacetate (DAF-2, 8 *μ*M, Sigma-Aldrich). Images were obtained with a fluorescence microscope (Nikon, Eclipse E600). Data were analyzed using the NIH Image J software.

### 2.11. RT-PCR Analysis

After an 8-week exercise, the thoracic aorta PVAT samples were collected from db/c mice, sedentary (nonexercised), and exercised db/db mice. The total RNAs were extracted by using Trizol reagent (Thermo Fisher Scientific) and used for uncoupling protein 1 (UCP-1) analysis. Reverse transcription (RT) reactions were performed using the PrimeScriptTM RT reagent kit (TaKaRa, Japan), and PCR reactions were conducted using SYBR Premix EX TaqTM II kit (TaKaRa, Japan). The relative expression level of each gene was normalized to U6 and calculated using the 2^-*ΔΔ*CT^ method.

### 2.12. ELISA Assays

The cytokine (adiponectin, IL-10, IFN-r, TNF-a, and IL-6) levels in PVAT samples from db/c, db/db exercised, and nonexercised mice were detected with specific antibody using the ELISA kits (BioLegend). The absorbance was measured at 450 nm by using a spectrometer (Bio-tek). Data was expressed as the fold of the level in db/c mice.

### 2.13. PVAT Collection and Culture

The PVAT samples were collected from the thoracic aorta of exercised db/db mice, rinsed using PBS, and then cut into small pieces. The samples were then cultured with DMEM supplemented with 10% exosomes-free FBS (System Bioscience's) overnight. On the next, the culture medium was collected, centrifuged at 300 g for 15 mins. Then, the supernatant was collected and considered as a PVAT-culture medium (PVAT-CM^db/db+E^) and used to treat the primary endothelial cells prior to adhesion assay.

### 2.14. Culture of Primary Aorta Endothelial Cells

The primary endothelial cells were isolated and cultured from the exercised and nonexercised db/db mice (*n* = 8/group) with slight modification [22]. In brief, the thoracic aorta was quickly removed and put it in ice-cold 1x PBS (sterile), then was gently flushed with ice-cold PBS to remove the blood, and the attached adipose tissue was removed. The aorta was immediately transferred to the endothelial growth medium and was cut into 1 mm rings. The aorta ring was then opened using a pair of microdissection scissors and placed lumen-side-down on growth factor reduced matrix (BD Bioscience) coated wells of a 6-well plate with 200 *μ*l endothelial growth medium and incubated in a standard incubator for 4 to 6 hrs. At the end of the day, the medium was added to cover the aortic segments. On the 4th day, the aortic segments were gently removed from the matrix without interrupting the growing endothelial cells. Cells were subcultured until they were 85-90% confluency.

### 2.15. Adhesion Assay of Primary Aorta Endothelial Cells

The adhesion assay was performed according to the published protocol with slight modification [23]. In brief, endothelial cells were seeded on gelatin-coated 24-well plates. HL60 cells (1 × 10^4^/well) labeled with acridine orange (Sigma) were then added into each well. One hour later, nonadherent HL60 cells were washed away twice with PBS. The number of HL60 cells adhering to endothelial cells was counted from 5 images of random fields under a fluorescence microscope (Nikon, Eclipse E600). Cell adhesion ability was defined as the average number of HL60 cells/field. Cells were collected for ICAM-1 and VCAM-1 analyses. Some endothelial cells were pretreated with PVAT-CM^db/db+E^ for 24 hrs prior to adding the HL60 cells.

### 2.16. Western Blotting

Immunoblotting was performed using standard techniques. Briefly, proteins from primary endothelial cell homogenates were separated on polyacrylamide-SDS gels and transferred onto PVDF membranes. Membranes were blocked; incubated with primary antibodies against VCAM-1 (1 : 100; Santa Cruz), ICAM-1 (1 : 100; Santa Cruz), and *β*-actin (1 : 4000, Sigma); and followed by a wash and then incubated with HRP-conjugated secondary antibodies. Immunodetection was carried out using ECL substrate (Bio-Rad).

### 2.17. Statistical Analysis

All data are presented as mean ± SEM. Two group comparsion was analyzed by student *t*-test. Multiple comparisons were analyzed by two-way ANOVA (SPSS version 16.0; SPSS, Chicago, IL, USA) followed by the Tukey test. For all tests, a *p* value < 0.05 was considered significant.

## 3. Results

### 3.1. Exercise Intervention Lows Body Weight Gain and Improves Glucose Tolerance and Insulin Sensitivity in db/db Mice

**Figure 1:**
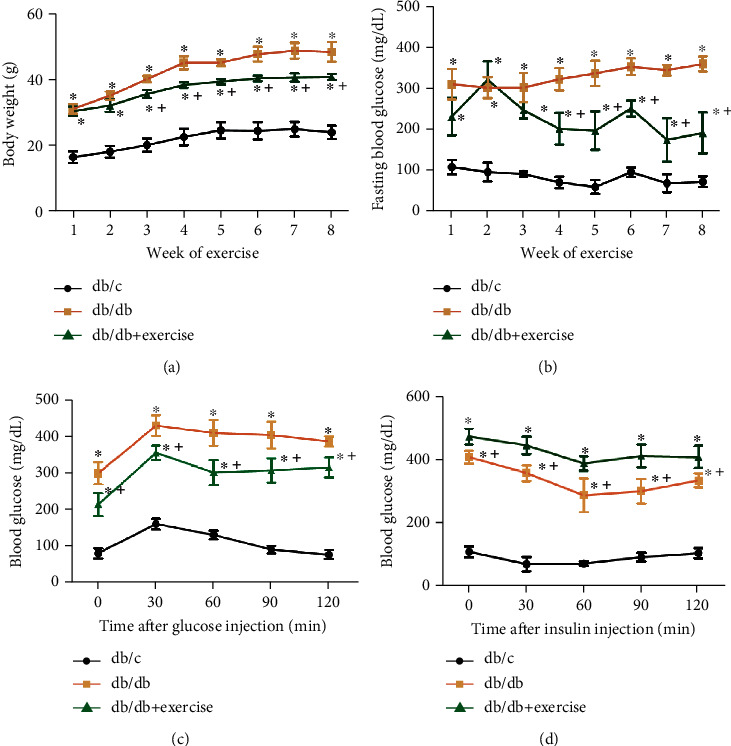
The effects of exercise intervention on body weight and metabolic characters of db/db mice. (a) Body weight of db/c, db/db, and db/db+exercise mice from week 1 to 8 of exercise. (b) Fasting blood glucose of the three groups during the 8-week exercise duration. (c, d) Blood glucose levels at 0, 30, 60, 90, and 120 mins after glucose or insulin injection. Db/db+exercise: db/db mice performed exercise. ^∗^*p* < 0.05, vs. db/c; + *p* < 0.05, vs. db/db. Data are expressed as mean ± SEM. *N* = 8/group.

As shown in Figure 1(a), the body weight of db/db mice was much higher than that in age-matched db/c mice. Started from the 3^rd^ week of exercise, the weekly body weight gain was significantly reduced in db/db mice as compared to that of the nonexercise ones. As expected, the fasting blood glucose of db/db mice was much higher than that of db/c mice. Exercise intervention significantly reduced the fasting blood glucose in db/db mice as compared to nonexercise db/db mice (Figure 1(b)).

We further studied the effects of an exercise intervention on metabolic function with both an IP-GTT and an IP-ITT after an 8-week exercise. The results of the IP-GTT demonstrated that the db/db mice cleared glucose less effectively than the db/c mice did after the i.p. glucose injection. Of note, eight-week exercise intervention improved glucose tolerance at all time-points after the i.p. glucose injection (Figure 1(c)). As expected, the results of IP-ITT showed that the diabetic db/db mice were significantly insulin intolerant compared to db/c control ones. Exercise intervention partially improved insulin intolerance in db/db mice (Figure 1(d)).

### 3.2. Exercise Intervention Increases the Number of Multilocular Brown Adipocytes and Upregulates the Levels of Brown Adipose Tissue Gene Marker UCP-1 in PVAT

**Figure 2:**
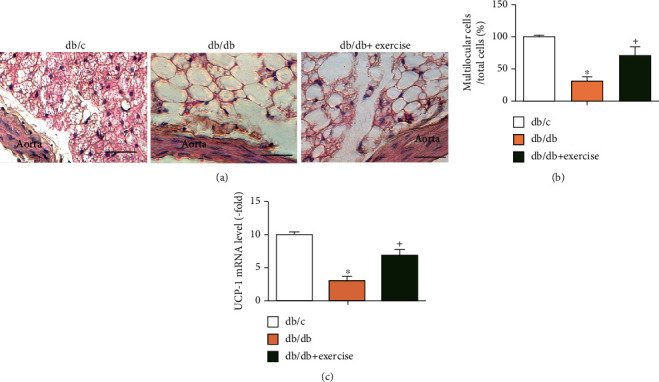
Analysis of the characteristics of PVAT. (a) Representative images of H&E staining of aorta PVAT, scale bar: 40 *μ*m. (b) Summarized data showing the percentage of multilocular cells in PVAT. (c) UCP-1 (white adipose tissue gene marker) mRNA levels in PVAT. Data expressed as mean ± SEM, *n* = 8/group. ^∗^*p* < 0.05, vs. db/c, + *p* < 0.05, vs. db/db.

As shown in Figure 2, there was a lower number of multilocular adipocytes in PVAT of db/db mice (~30%) than that in db/c mice (~100%), suggesting the adipose tissue status is changed in diabetic condition. Exercise intervention increased the number of multilocular adipocytes in PVAT of db/db mice (~60%) as compared to that in sedentary (nonexercise) db/db mice. We further confirmed the expression of brown adipose tissue gene (UCP-1) and found that UCP-1 level was upregulated in exercised db/db mice.

### 3.3. Exercise Intervention Promotes Macrophage Polarization from M1 to M2 in PVAT

**Figure 3:**
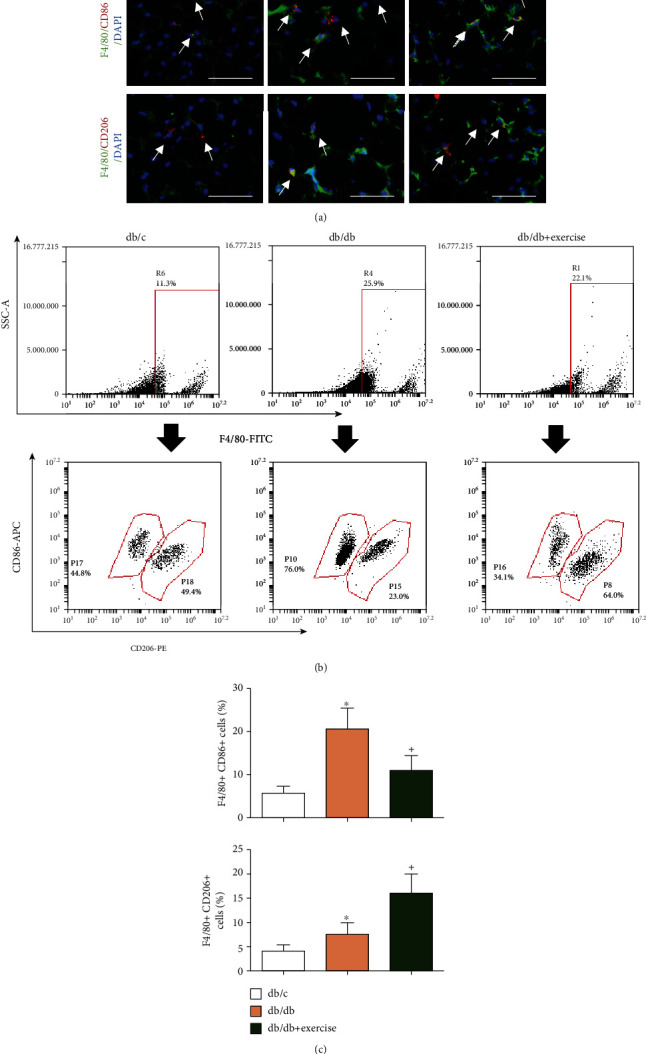
The effects of exercise intervention on macrophage polarization in PVAT. (a) Representative images showing the double-positive staining of F4/80 and CD86, F4/80 and CD206. Scale bar: 50 *μ*m. White arrows indicate the double-positive cells. Green: F4/80; red: CD86 or CD206; blue: nucleus counterstained by DAPI. (b) Representative flow plot showing the percentage of M1 and M2 macrophages. The gate in the upper panels shows the positive events of F4/80, the down panels showing the double-positive expression of F4/80 and CD86 or CD206. (c, d) Summarized data show the percentage of M1 (F4/80+CD86+) and M2 (F4/80+CD206+) cells in PVAT in the three groups. Db/db+exercise: db/db mice performed exercise. ^∗^*p* < 0.05, vs. db/c; + *p* < 0.05, vs. db/db. Data are expressed as mean ± SEM. *N* = 8/group.

As revealed by immunofluorescence staining and flow cytometry analyses (Figure 3), there was a higher percentage of M1 macrophages (F4/80+CD86+ cells) in the PVAT of db/db mice than that in db/c mice (~20% and~5% for db/db and db/c, respectively). After 8-week exercise, less M1 cells (~11% for exercised db/db) whereas more M2 cells (~7% and~15% for sedentary db/db and exercised db/db, respectively) were detected in the PVAT of exercised db/db mice as compared to that in the nonexercised ones.

### 3.4. Exercise Intervention Decreases Proinflammatory Cytokine whereas Increases Anti-inflammatory Cytokine and Adipokine Levels in PVAT of db/db Mice

We examined the levels of inflammatory-related cytokines and adiponectin (a type of adipokine) in PVAT in db/c, exercised, and nonexercise db/db mice. Our data (Table 1) showed that the nonexercised db/db mice had higher levels of proinflammatory cytokines such as IFN-r, TNF-a, and IL-6 and lower levels of an anti-inflammatory cytokine such as IL-10 and a lower level of adiponectin in PVAT than those in db/c mice. Eight-week treadmill exercise significantly reduced the proinflammatory but raised the anti-inflammatory cytokine and adiponectin levels in PVAT in db/db mice.

### 3.5. Exercise Intervention Alleviates Oxidative Stress in PVAT and Aorta, as well as Improves Endothelial NO Production in Aorta

**Figure 4:**
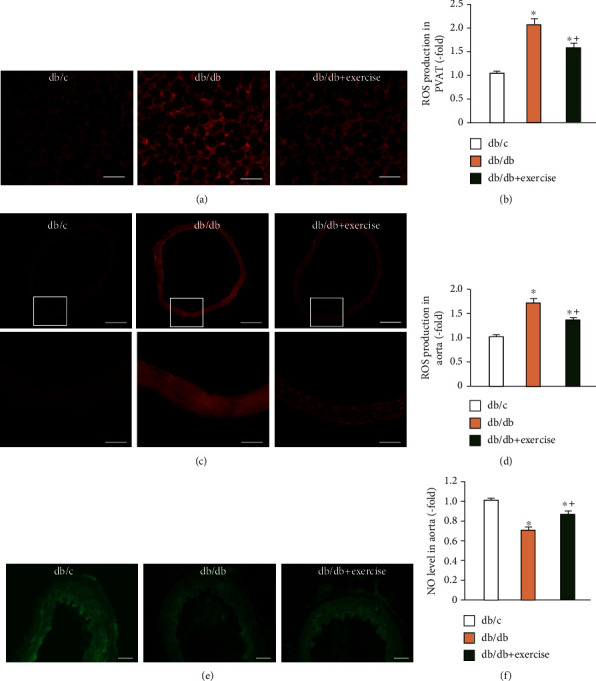
Analysis of ROS and/or NO level in PVAT and aorta. (a, b) Representative images and summarized data showing ROS level in PVAT in db/c, db/db, and db/db+exercise mice after 8-week exercise. Scale bar: 50 *μ*m. (c) Representative images showing ROS expression in the aorta; bottom panels are enlarged from the relative box areas of upper panels. Scale bar in the aorta: 200 *μ*m (upper panels) and 50 *μ*m (bottom panels). (d) Summarized data of the ROS level in the aorta in the three groups. (e) Representative images showing NO expression in the aorta. Scale bar in aorta: 40 *μ*m. (f) NO level in aorta from db/c, db/db, and db/db+exercise mice after 8-week exercise; Db/db+exercise: db/db mice performed exercise. ^∗^*p* < 0.05, vs. db/c; + *p* < 0.05, vs. db/db. Data are expressed as mean ± SEM. *N* = 8/group.

The oxidative stress in PVAT and aorta was detected by DHE staining. As shown in Figures 4(a)–4(d), ROS generation in PVAT was significantly higher in db/db mice than that in db/c mice. After exercise, the ROS level was remarkably reduced in PVAT of db/db mice. Similarly, the ROS level in the aorta was downregulated in the exercised db/db mice when compared to the nonexercised db/db mice. The NO level was significantly decreased in the aorta of db/db as compared to the db/c mice. Eight-week treadmill exercise intervention raised NO production in db/db mice (Figures 4(e) and 4(f)).

### 3.6. Exercise Intervention Decreases Endothelial Adhesion Ability Associating with Downregulations of VCAM-1 and ICAM-1

**Figure 5:**
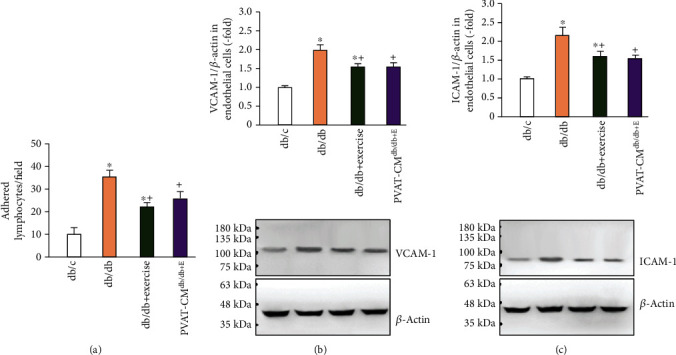
The effects of exercise intervention on aorta endothelial cell inflammation. (a) The adhesion ability of primary aorta endothelial cells to lymphocytes. (b, c) Protein levels of ICAM-1 and VCAM-1 in the primary aorta endothelial cells from the three mouse groups and pre-treated by PVAT-CM. Db/db+exercise: endothelial cells cultured from exercised db/db mice. PVAT-CM^db/db+E^: endothelial cells cultured from db/db mice were pretreated with the culture medium of PVAT isolated from exercised db/db mice. ^∗^*p* < 0.05, vs. db/c; + *p* < 0.05, vs. db/db. Data are expressed as mean ± SEM. *N* = 8/group.

From the results of adhesion assay (Figure 5(a)), there were fewer lymphocytes adhered to endothelial cells cultured from db/db mice than those from db/c mice (10 ± 4 and 34 ± 3 lymphocytes/field for db/c and db/db, respectively). Exercise decreased the adhesion ability of endothelial cells in db/db mice (34 ± 3 and 22 ± 2 lymphocytes/field for db/db and db/db+exercise, respectively). Of note, the culture medium from PVAT of the exercised db/db mice (PVAT-CM^db/db+E^) could also significantly decrease the adhesion ability of endothelial cells from db/db mice (*p* < 0.05, vs. db/db).

ICAM-1 and VCAM-1 are two critical adherent molecules released by endothelial cells which could promote leukocyte adhesion [24]. We further conducted a western blot to assess their expressions. Our results (Figures 5(b) and 5(c)) show that there were high levels of both ICAM-1 and VCAM-1 in the endothelial cells cultured from db/db mice as compared to those from the db/c mice. Exercise downregulated the expressions of ICAM-1 and VCAM-1 in aorta endothelial cells in db/db mice. Similarly, those cells pretreated by PVAT-CM also had lower expressions of ICAM-1 and VCAM-1 (*p* < 0.05, vs. db/db).

## 4. Discussion

In this study, we have demonstrated that moderate treadmill exercise can improve endothelial function in diabetes, associating with the alleviation of inflammation and oxidative stress in PVAT. Of interest, the in vitro study showed that the culture medium of PVAT collected from exercised db/db mice could reduce the adhesion ability of primary endothelial cells of db/db mice.

Increased physical activity is routinely recommended in the management of human T2D and is believed to improve glycemic control and plasma lipids, while simultaneously decreasing insulin resistance and body weight [25]. In this study, our data showed that moderate treadmill exercise intervention could not only reduce body weight but also lower the fasting blood glucose level in db/db mice, indicating the beneficial effects of exercise on metabolism in diabetes. This is consistent with previous evidence showing that exercise can decrease risk factors of vascular functions such as blood glucose and body mass index [25]. Hyperglycemia in diabetes results in oxidative stress and low-grade chronic inflammation which are implicated in diabetes-associated vascular complications. Therefore, controlling the blood glucose level could prevent long-term vascular complications. Glucose intolerance is an umbrella term for metabolic conditions in which sugar is not absorbed properly through the intestines into the bloodstream, resulting in hyperglycemia. Besides, a resistance to the hormone insulin also results in increasing blood sugar. With insulin resistance, the cells do not respond normally to insulin. Previous evidence shows that exercise can help to alleviate insulin resistance [26]. Here, we demonstrated that the moderate exercise regimen improved glucose tolerance and insulin sensitivity in db/db mice.

Brown adipose tissue has been suggested to be more vascular and more metabolically active in comparison with white adipose tissue [27]. As expected, in diabetic condition, thoracic aorta PVAT becomes a complex of white and brown adipocyte tissue as evidenced by a lower percentage of multilocular adipocytes and decreased UCP-1 level. To investigate whether exercise intervention can affect the adipocyte type in PVAT, we conducted HE staining and found that there was increased brown adipose tissue in the PVAT of exercised diabetic mice as evidenced by an increasing number of multilocular adipocytes and the raised UCP-1 expression in PVAT. These findings suggest that an 8-week exercise regimen can modulate the adipocyte status in PVAT. Of note, as demonstrated by previous studies, brown adipose tissue represents approximately 5% of the total adipose tissue in the general population [28, 29]. Various factors such as age, sex, and diabetic control status might impact the mass and function of brown adipose tissue in humans [30–32]. Although previous reports have demonstrated that exercise training can elicit beneficial effects on type 2 diabetic patients [33], whether the brown adipose tissue such as aorta PVAT in human subject responses to moderate exercise regimen requires more investigation.

Here, in the diabetic mouse, we found that there was an increased percentage of proinflammatory M1-macrophages in PVAT, reflecting an inflammatory status in diabetic PVAT. As we know, macrophages are one type of immune cells to regulate PVAT inflammation. Previous studies have shown that macrophages could accumulate in PVAT during hypercholesterolemia and hypertension [34, 35]. As indicated by previous studies, macrophage polarization from M1-macrophages to M2-macrophages confers vast phenotypic and functional plasticity, allowing them to act as pro-inflammatory and anti-inflammatory agents [36]. Kawanishi and colleagues have shown that exercise alleviates inflammation in the gonadal adipose tissue in high-fat diet-fed mice [16, 17]. In the present study, we hypothesized that the 8-week treadmill exercise regimen can modulate macrophage polarization in PVAT of the T2D mice. As expected, we found that there was a higher number of M2-macrophages in the PVAT of exercised db/db mice as analyzed by immunofluorescence staining and flow cytometry analysis. Consistently, our data showed that the cytokine profiles were significantly changed in PVAT as evidenced by the upregulated expressions of anti-inflammatory cytokine IL-10 and adiponectin and downregulated proinflammatory cytokine (TNF-a, IFN-r, and IL-4) levels. All of these data indicate that exercise intervention alleviated the inflammatory status in PVAT by which might contribute to vascular function improvement.

It is generally accepted that oxidative stress can lead to cell and tissue injury and has a fundamental role in vascular dysfunction [10]. Oxidative stress arises from an imbalance when the production of ROS exceeds that of endogenous antioxidant enzymes. Excessive ROS such as superoxide is highly reactive with NO and decreases the production and bioavailability of NO in the vasculature [37]. PVAT-derived ROS has been shown to be implicated in endothelial dysfunction in diet-induced obese C57BL/6 mice [38]. Hence, we investigated whether the moderate treadmill exercise regimen could modulate oxidative stress in both PVAT and aorta. Specifically, we found that ROS generation was decreased in both PVAT and aorta in exercised diabetic mice which might contribute to the alterations of production and bioavailability of NO in the aorta. As revealed by previous studies, basal NO level plays an important role in maintaining vessel tone [39]. An increase in NO production underlies the vasorelaxant action of multiple vasorelaxant mediators. Here, we found that an 8-week exercise intervention could raise NO level in the aorta of diabetic db/db mouse, providing evidence to support our assumption that exercise could improve the function of the aorta. Whether the stimulation of acetylcholine, a potent vasodilator, could induce NO level change in the aorta endothelium cells in exercised db/db mice needs further investigation. In addition, Roberto and colleagues have revealed that intracellular Ca^2+^ signaling plays an important role in modulating endothelial cell physiology such as NO release, ROS production, and adhesion molecule secretion [40]. However, the Ca^2+^ signaling machinery is impaired in situ vascular endothelial cells from diabetic rodents [40, 41]. In the present study, although we did not detect the possible changes of Ca^2+^ signaling in vascular endothelial cells before and after exercise intervention in the three groups of mice, our data is partially supported by previous reports showing that physical exercise could enhance multiple mechanisms of artery relaxation including activating the Ca^2+^ signaling machinery [42, 43]. Meanwhile, we also found that exercise intervention significantly raised the adiponectin level in PVAT of db/db mice. This finding is supported by a previous report demonstrating that PVAT may be involved in cardiovascular function through the release of adipokines to elicit intracellular Ca2+ signals in vascular endothelial cells [44].

To further confirm the vascular endothelium status, we conducted in vitro experiment to determine the adhesion ability of primary aorta endothelial cells. We found that the expressions of proinflammatory adhesion molecules (ICAM-1 and VCAM-1) were downregulated in primary aorta endothelial cells isolated from the exercised db/db mice, which lead to a decreased adherence of lymphocyte to endothelial cells in vitro. This data also supports our results showing the decreased inflammation in the aorta after exercise.

At last but not least, we measured the function of PVAT in vitro by culturing the PVAT from animals and coincubated the culture medium of PVAT with the primary endothelial cells. Of note, we found that the culture medium of PVAT from exercise diabetic db/db mice also reduced ICAM-1 and VCAM-1 expressions in the primary endothelial cells. The observed effects might be contributed to the paracrine mechanism of PVAT such as cytokines or extracellular vesicles that are novel intercellular communicators [45] which need further investigation.

In conclusion, our data have demonstrated that exercise intervention can alleviate inflammation and oxidative stress of PVAT which contribute to improving the vascular function in T2D mice.

## Figures and Tables

**Table 1:** Analysis of the proinflammatory and anti-inflammatory cytokine levels in PVAT. Cytokine levels in PVAT of db/c and db/db mice.

	db/c	db/db	db/db+exercise
Adiponectin level (-fold)	1 ± 0.05	0.45 ± 0.07^∗^	0.7 ± 0.08^∗+^
IL-10 level (-fold)	1 ± 0.06	0.5 ± 0.08^∗^	0.8 ± 0.05^∗+^
IFN-r level (-fold)	1 ± 0.08	1.8 ± 0.1^∗^	1.4 ± 0.08^∗+^
TNF-a level (-fold)	1 ± 0.1	2.2 ± 0.2^∗^	1.5 ± 0.1^∗+^
IL-6 level (-fold)	1 ± 0.13	2.5 ± 0.3^∗^	1.8 ± 0.2^∗+^

Data are expressed as mean ± SEM. ^∗^*p* < 0.05, vs. db/c; + *p* < 0.05, vs. db/db. *N* = 8/group.

## Data Availability

The data that support the findings of this study are available from the corresponding author upon reasonable request.
